# Supplementary material for: Psychoeducation Improved Illness Perception and Expressed Emotion of Family Caregivers of Patients with Schizophrenia
Source: Int J Environ Res Public Health. 2021 Jul 15;18(14):7522. doi: 10.3390/ijerph18147522 (PMC8307551; doi:10.3390/ijerph18147522)
Supplement: Supplementary file 1 [file ijerph-18-07522-s001.zip › ijerph-1248534-supplementary.pdf]

**Supplementary Table S1.** Kaiser-Meyer-Olkin (KMO) measure of sampling adequacy for IPQS-R.

| IPQS-R Subscales | KMO |
|---|---|
| Identity | - |
| Cause Items | 0.76 |
| Timeline Acute | 0.64 |
| Timeline Cyclical | 0.56 |
| Consequence – Patient | 0.66 |
| Consequence – Relatives | 0.73 |
| Personal Control – Patient | 0.55 |
| Personal Control – Relatives | 0.6 |
| Personal Blame – Patient | 0.64 |
| Personal Blame – Relatives | 0.62 |
| Treatment Control | 0.54 |
| Illness Coherence | 0.72 |
| Emotional Representation | 0.59 |

**Supplementary Table S2.** Inter-subscale Pearson's correlations for the IPQS (N = 128). * showed significant correlation between subscales items; $p < 0.05$.

| IPQS Subscales | Identity | Timeline acute | Timeline cyclical | Consequences | Personal control | Personal blame | Treatment control | Coherence |
|---|---|---|---|---|---|---|---|---|
| Timeline acute | −0.171 | | | | | | | |
| Timeline cyclical | 0.014 | −0.091 | | | | | | |
| Consequences | 0.393 * | −0.298 * | 0.064 | | | | | |
| Personal control | −0.275 * | 0.375 * | −0.083 | −0.690 * | | | | |
| Personal blame | 0.030 | −0.053 | 0.047 | 0.293 * | −0.252 * | | | |
| Treatment control | −0.333 * | 0.312 * | 0.075 | −0.595 * | −0.533 * | −0.210 * | | |
| Coherence | 0.230 * | −0.259 * | 0.023 | 0.614 * | −0.611 * | 0.216 * | −0.475 * | |
| Emotional representation | 0.359 * | −0.361 * | 0.089 | 0.618 * | −0.652 * | 0.326 * | −0.647 * | 0.748 * |
